# Diagnostic accuracy of 18F-FDG-PET in abdominal sepsis in rats ^1^

**DOI:** 10.1590/s0102-865020200050000005

**Published:** 2020-06-22

**Authors:** Ítalo Medeiros Azevedo, Robson Macedo, Keyla Borges Ferreira Rocha, Cláudia Nunes Oliveira, Aldo Cunha Medeiros

**Affiliations:** I Fellow PhD degree, Postgraduate Program in Health Sciences, Universidade Federal do Rio Grande do Norte (UFRN), Natal-RN, Brazil. Technical procedures, acquisition of data, statistics analysis, critical revision.; II PhD, Full Professor, Nucleus of Experimental Surgery, UFRN, Natal-RN, Brazil. Conception, design, intellectual and scientific content of the study; critical revision; final approval.

**Keywords:** Abdomen, Sepsis, Diagnosis, Fluorodeoxyglucose F18, Positron Emission Tomography Computed Tomography, ROC Curve, Rats

## Abstract

**Purpose:**

The objective of this study was to investigate the accuracy of 18F-FDG-PET in the diagnosis of multibacterial abdominal sepsis by cecum ligation and puncture (CLP) in rats.

**Methods:**

Adult Wistar rats ( *Rattus norvegicus* ), weighing 227±35g, were allocated into a sepsis group by CLP (n=10) and sham group (n=10). 18F-FDG-PET using microPET was performed on all rats after 24 hours.

**Results:**

All animals survived for postoperative 24h. The abdomen/liver ratio of the standardized uptake value (SUV) percentage was significantly higher in the sepsis group than in the sham (p=0.004). The ROC curve showed an accuracy of 18F-FDG-PET to detect abdominal sepsis of 88.9% (p=0.001), sensitivity of 90% and specificity of 88.9%. When a cut-off point of 79% of the ratio between the SUV on the abdominal region and liver was established, the sensitivity was 90%, specificity of 88.9%; positive and negative predictive values of 90.0% and 88.9%, respectively.

**Conclusions:**

The diagnostic accuracy of 18F-FDG-PET in rats with abdominal sepsis was significantly high. It was also demonstrated the predictive ability of the abdomen/liver SUV ratio to diagnose abdominal sepsis. These findings may have implications for the clinical setting, locating septic foci with PETscan.

## Introduction

Abdominal sepsis results in the systemic inflammatory response syndrome, as an inflammatory response due to intra-abdominal infection, trauma, and various diseases^1^ . Sepsis is considered to involve physiological changes in the body towards high metabolism. Characteristic changes include excessive metabolism of proteins and lipids, increased energy consumption, negative nitrogen balance, excessive production of glycogen levels and hyperglycemia^2^ . Hyperglycemia is an important causal factor for increased metabolism in sepsis and is responsible for high morbimortality^3^ .

The diagnostic impact of 18F-FDG-PET needs to be assessed in preclinical and clinical studies, as data on the diagnostic accuracy of 18F-FDG-PET in the detection of abdominal sepsis are currently scarce. It is an important imaging exam that can provide information about anatomy, physiology and pathology. It is increasingly used for the diagnosis and follow-up of cancer, and more recently, infection and inflammatory diseases^4^ . 18F-FDG-PET has shown promising results in patients with fever of unknown origin^5^ and has gained visibility in the identification of inflammatory foci in soft tissues, bone and vascular structures^6 , 7^ .

In cases of cancer, infection and inflammation, the compromised cells have high metabolic activity and intense glycolysis, because of the high content of glucose transport proteins and the increase in hexokinase activity^8 , 9^ . Within the cell, the transporters are located in the plasma membrane and in intracellular vesicles. After stimulation, translocation of the transporters to the cell membrane occurs. Lymphocytes demonstrate this transport effect a few minutes after stimulation^10^ , which may explain the usefulness of 18F-FDG-PET in the early diagnosis of inflammation and infection and the importance of this imaging test for the diagnosis of septic foci. The ease of making a full body image, anatomical location, metabolic information and high spatial resolution, are some of the advantages offered by the 18F-FDG-PET compared to other nuclear medicine and conventional imaging techniques. The indications for 18F-FDG-PET in the diagnosis of infectious diseases are well established in endocarditis, tuberculosis, osteomyelitis, vasculitis, infections of vascular prostheses and fever of obscure origin^11^ . In addition, 18F-FDG-PET uses a low radiation dose and offers high spatial and contrast resolution. The potential advantage of 18F-FDG-PET is to detect signs of abdominal sepsis before tissue necrosis and definite abscess occur^12^ .

The aim of this study was to investigate the accuracy of 18F-FDG-PET in the diagnosis of multibacterial abdominal sepsis by cecum ligation and puncture (CLP) in rats.

## Methods

The project was submitted to the institutional Animal Use Ethics Committee, and it was approved under protocol 003/2015. All experimental procedures were performed based on the guidelines of Brazilian Law No. 11,794/08.

Adult Wistar rats ( *Rattus norvegicus* ) weighing 227±35g, from the vivarium of the Health Sciences Center of the Federal University of Rio Grande do Norte, Brazil, were used. The rats were acclimatized for 1 week before the experiment under standard housing, including room temperature 22-24 °C, relative humidity 40% and 12-hour light-dark cycle in polypropylene cages. They had *ad libitum* access to the rodent diet (Prevence®) and water.

### Experimental design

Twenty animals were used, distributed in 2 groups: Sham group (n=10) and sepsis group (n=10).

### Surgical procedures

All rats were weighed before surgery. After anesthesia with xylazine (10 mg/kg) associated with ketamine (70 mg/kg) intraperitoneal, epilation of the abdominal wall and antisepsis with 70% alcohol were done. Fixation of the animals on the operating table and placement of sterile fields on the abdomen were performed. Sham group rats were submitted to a median laparotomy of 4 cm and wound suture. Sepsis group rats were submitted to the cecum ligation and puncture (CLP) using 3-0 polyester suture, 1 cm away from the ileum penetration, proceeding four punctures of the cecum with a sterile 25/8F needle. Then, the cecum was replaced in the abdominal cavity, closing the abdominal wall and skin with 4-0 nylon suture. Postoperative pain was controlled with meperidine at a dose of 10mg/kg subcutaneously immediately after the surgical procedure and every 12 hours.

### Survival and weighing

The animals were kept under observation in individual polypropylene cages for 24 hours, and their survival time was recorded in hours and minutes. After the observation period, the rats were weighed again.

### Blood glucose measurement

Twenty-four hours after sepsis induction, blood was collected through tail vein of the anesthetized animals, immediately before imaging exams, for blood glucose measurement, using the OneTouch-ultra equipment, Johnson-Johnson, Sao Paulo, Brazil.

### 18-FDG-PETscan imaging

At the end of postoperative 24-hour, the animals remained on zero diet for 6 hours before the tracer injection (18F-FDG), and were anesthetized with the technique described above. The 18F-FDG was purchased from the Energy and Nuclear Research Institute, Sao Paulo, Brazil. The dose with an activity of 26.2±4.46 MBq was injected into the femoral vein surgically identified, using a surgical microscope (DFV, Sao Paulo, Brazil). After 30 minutes, three-dimensional 18F-FDG-PET images were captured from the animals, using the Bruker Albira microPET Preclinical Image System (New Haven, CT, USA). Each animal was individually conditioned in a specific rack of the equipment, and data of radiopharmaceutical dosage information was entered into the machine. The image capture followed a unique pattern defined in the equipment protocol, whose method considered the capture ring width (40 mm) with sweeping of the entire body of the animal (except tail), being 10 min in each passage, in 40 mm cuts , in a total of 5 passes, making up 50 min of examination for each animal.

All images were analyzed independently by a researcher experienced in nuclear medicine (R.M.) at Albira microPET. For quantitative measurements of the metabolic activity of 18F-FDG in all rats, the standardized uptake value (SUV) was calculated using the embedded software (PMOD). SUV was the fraction of the activity administered of 18F-FDG per milliliter of tissue, multiplied by the rats weight, considering the tissues and regions of interest (ROI) of the areas under study.

### Macroscopic examination of the abdomen

After the imaging was completed, with the animals still anesthetized, euthanasia was performed with an intracardiac overdose of sodium thiopental (100mg/kg). Then, a median laparotomy was performed, for wide access to the abdominal organs looking for septic foci and other alterations resulting from sepsis. The abdominal cavity was photographed using a Sony DSC-HX100V camera, 16.2 megapixels, Japan. The photographic images were stored for correlation with the 18F-FDG-PET images.

### Statistical analysis

To define the SUV’s predictive capacity, we sought to find an ideal cutoff point for the SUV in the region of interest, in animals of both groups. The statistical method adopted was the Receiver Operating Characteristic Curve (ROC Curve), which was generated by calculating the sensitivity, specificity and accuracy. After determining the cutoff point, the association was verified by Fisher’s exact test.

Quantitative variables were expressed as mean ± standard deviation. To test whether the hypothesis of difference between SUV measurements, glycemia level and animal weight were statistically different between groups, Student’s t-parametric and Mann-Whitney non-parametric tests were adopted. For all tests, the 95% confidence interval was adopted. The IBM SPSS®21 Statistics package was used.

## Results

All animals survived until the end of the observation period. At this time, the weight of the animals in the sepsis group was lower (224.0±29.35g) than the sham (234.5 ± 36.57g), but the difference was not significant (p = 0.503). Table 1 summarizes the average values and variability of the injected dose of 18F-FDG (MBq), weight (g) of the animals and blood glucose (mg / dL), in addition to inferential tests.

**Table 1:** Descriptive and inferential values of 18F-FDG, weight and blood glucose of animals in both groups.

Parameters	Groups		p-value

	Sham	Sepsis	
18F-FDG (MBq)	26.7±4.06	25.9±5.38	0.510^1^
Weight (g)	234.5±36.57	224.0±29.35	0.503^2^
Glycemia (mg/dL)	226.6±34.3	104.5±33.74	<0.001^2^

Mean ± standard deviation

1. Mann-Whitney non-parametric test; 2. Student t test

The data in Table 1 indicate that there was no difference between the injected dose of 18F-FDG in the animals in the sham group compared to the sepsis group (p = 0.510). There was also no difference among the animals’ weights, when comparing groups (p=0.503). The blood glucose level was significantly lower (p <0.001) in the sepsis group, when compared to the sham group.

The data for the quantification of 18F-FDG activity through the SUV measured in the different organs, as well as the ratio of the percentage of activity of the abdomen/ liver, are summarized in Table 2 .

**Table 2:** SUV values of 18F-FDG and respective inferential tests, by group.

SUV	Group		p-value

	Sham	Sepsis	
Brain (%ID/cm^3^)	6.92±6.04	11.93±6.11	0.041^1^
Heart (%ID/cm^3^)	0.52±0.28	0.43±0.12	0.568^1^
Liver (%ID/cm^3^)	0.31±0.16	0.54±0.15	0.005^2^
Abdominal region (%ID/cm^3^)	0.13±0.06	0.67±0.18	0.009^1^
Abdomen/liver (%)	87.48±129.96	129.23±39.89	0.004^1^

Mean ± standard deviation

1. Mann-Whitney non-parametric test; 2. Student t test

ID: Injected Dose; SUV: Standardized Uptake Value

In the heart, there was no significant difference between the SUV of the study groups (p> 0.05). However, the SUV in the brain, liver and abdominal region was significantly higher in the sepsis group than in the sham group (p <0.05). The ratio of the percentage of SUV captured in the abdomen and liver was significantly higher in the sepsis group than in the sham group (p = 0.004)

### Results for the percentage of 18f-fdg uptake higher than 79% in the abdomen

The area under the receiver operating characteristic curve (ROC curve) showed an accuracy of 88.9% (p = 0.001, CI 68.4-100%). The 79% cutoff point for 18F-FDG uptake in the abdomen with respect to the liver was established to verify the predictive capacity of this value ( Table 3 ).

**Table 3:** Association between the presence of sepsis and the percentage of SUV, abdomen/liver, considering the cutoff point of 79%.

SUV Abdomen/liver (%)	SEPSIS				p-value^1^

	YES		NO		

	N	%	N	%	
>=79	9	90.0	1	10.0	0.001
<79	1	11.1	8	88.9	

1. Fisher’s exact test; OR: Odds Ratio

Table 3 shows that there is an association between the percentage of the SUV ratio arbitrated through the ROC Curve (> = 79.0%) and the presence of sepsis (p = 0.001). The sensitivity was 90%, specificity 88.9%, positive predictive value (PPV) 90.0% and the negative predictive value (NPV) 88.9%. Additionally, the positive and negative probability ratios were calculated. The values were 8.2 and 0.11, thus indicating the moderate predictive accuracy of the arbitrated cut-off point.

**Figure 1:**
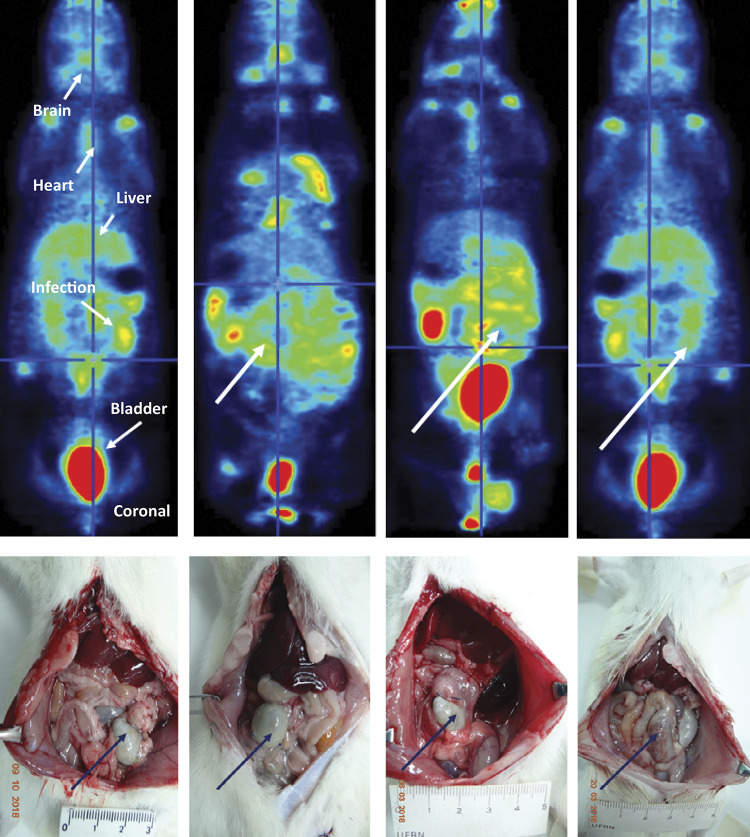
18F-FDG-PET scan image of four rats from the sepsis group. The first PETscan on the left contains arrows that identify the radioactive uptake images of brain, heart, liver, foci of abdominal infection and urinary bladder. White arrows show PETscan abdominal septic foci images and black arrows show septic cecum in the open abdominal cavity of respective rats.

Figure 1 illustrates 18F-FDG-PET of four rats of the sepsis group, and respective photographs of the surgically open abdominal cavities. It is demonstrated increased F18-FDG uptake in several areas in the abdomen, suggestive of abdominal sepsis. The white arrows indicate areas of F18-FDG uptake in the abdomen, which coincide with the position of the cecum submitted to ligation and puncture and areas of hyperemia in the abdominal cavities on the lower photos (black arrows).

**Figure 2:**
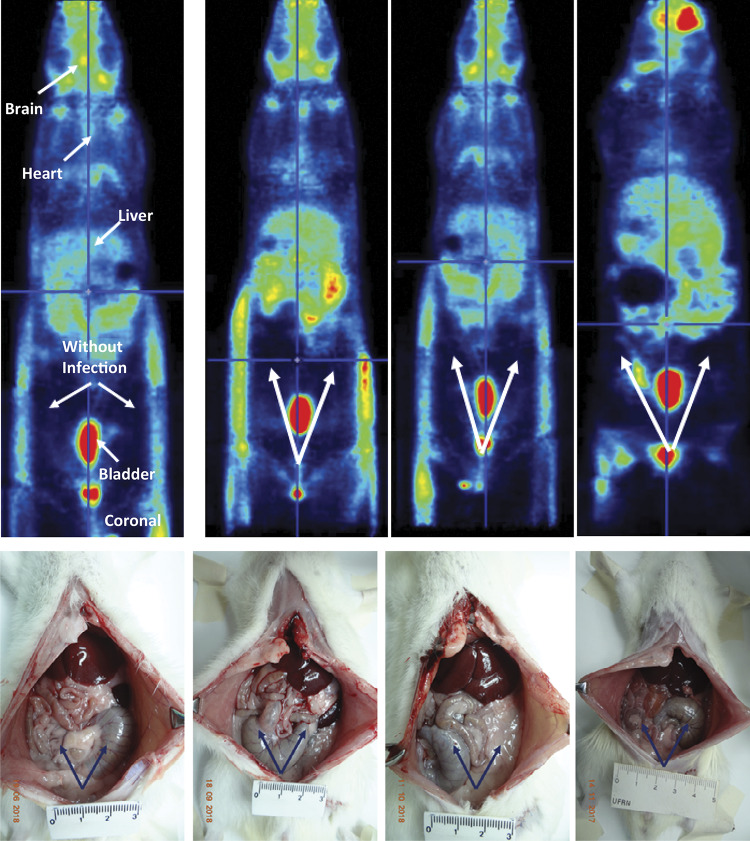
Representative images of 18F-FDG-PETscan and respective photographs of the abdominal cavity of four rats from the sham group. The first PETscan on the left contains arrows that identify the radioactive uptake images of some organs (brain, heart, liver, foci of abdominal infection and urinary bladder).

18F-FDG-PET images of four rats from the sham group are showed in Figure 2 . No 18F-FDG uptake is observed in the organs of infrahepatic abdominal cavity (white arrows). The respective inferior photographs were obtained immediately after the imaging exams, and all are showing normal cecum and intestines in the abdominal cavity (black arrows).

## Discussion

The present study investigated the action of 18F-FDG-PET on the diagnostic accuracy of abdominal sepsis in rats. In this study, blood glucose was measured before the 18F-FDG-PET was performed. Hyperglycemia is frequently seen in septic patients, associated with the great severity of the disease, with high morbidity and mortality. In response to an acute injury, high levels of serum hormones, such as glucocorticoids and catecholamines, are released, increasing liver gluconeogenesis and insulin resistance. In addition, during sepsis, pro-inflammatory cytokines participate in its pathogenesis^13^ . Several studies have shown that hyperglycemia can result in reduced FDG uptake in tissues, presumably due to the competitive inhibition of Glut-1 and 3 and hexokinase activity^14 - 16^ .

Paradoxically, data from this study showed that, 24 hours after CLP, blood glucose levels in septic rats were significantly lower than in sham rats. It was established the following hypothesis to explain this finding: the feeding behavior of the animals was substantially reduced after the induction of sepsis. As the rats have a high active metabolism, the energy required by them after trauma and sepsis was expected to be significantly elevated. Thus, overnight fasting may have caused significant hypoglycemia compared to sham rats. This possibly caused a reduction in metabolic capacity after severe sepsis. In fact, the septic animals in the present study were clinically dehydrated and with weight loss 24 hours after sepsis induction. In this study the CLP sepsis model was used, following the technique of previous experimental reports^17 , 18^ .

18F-FDG is a radioactive marker similar to glucose, which has been used for in vivo studies of changes in the metabolic capacity of glucose in tissues and organs in humans and animals. 18F-FDG is transported to cells by glucose transporters located on the cell membrane, then phosphorylated into 18F-2’-FDG-6 by hexokinase and are retained in the cells. 18F-FDG levels depend on the cells ability to transport and metabolize glucose. Evidence shows that high uptake of 18F-FDG does not only occur in neoplasms, but it is also present in sepsis^19^ .

Malignant cells show high metabolic activity and intense glycolysis, because of the high content of glucose transport proteins or the increase in hexokinase activity^8 , 9^ . The same principle applies to sepsis and inflammation. The following mechanisms play an important role: (1) a cascade of inflammatory reaction, and a consequent increase in glycolysis^20^ ; (2) increased number of glucose transporters into cells; and (3) great affinity of glucose transporters for 18F-FDG^10 , 21^ . Glucose transporters mediate the transport of glucose and FDG to cells. After sepsis, translocation of the transporters to the cell occurs, and lymphocytes have this transport effect a few minutes after challenge^22^ . These mechanisms may explain the usefulness of 18F-FDG-PET in the early diagnosis of inflammation and infection and the importance of this imaging test for the diagnosis of septic foci.

In this study, the liver was used as the standard image to compare with the images of the abdominal organs affected by sepsis. 18F-FDG is dynamically exchanged between hepatocytes and blood, resulting in a hepatic 18F-FDG concentration approximately equal to blood concentration^23^ . It has been recommended that, in basic research with small animals, as in this study, the liver is the ideal organ for monitoring the biodistribution of activity in blood, tissues and organs^23^ . The ROC curve, calculated from the ratio between the SUV of the abdominal region and the liver, showed high accuracy, demonstrating that the 18F-FDG-PET had a high sensitivity, specificity, positive and negative predictive values, in addition to demonstrating moderate probability accuracy. These findings are in agreement with the data reported by other authors who used the ROC curve to assess the role 18F-FDG in the detection of infection, by calculating the accuracy by the SUVmax.^24 , 25^ . A limitation of this study was that we did not use fused images 18F-FDG-PET/CT, because our equipment is only a microPET. The FDG-PET/CT fused image would bring together anatomy and metabolism, thus offering an interesting tool to locate abdominal septic foci.

The good results in identifying the foci of sepsis using PET observed in vivo is presumably a combination of local hyperemia and vascular leakage^26^ . We believe that PETscan could be applied for the diagnosis of peritonitis, liver abscess, pelvic abscess, psoas abscess and other abdominal septic focus. Given the accuracy of 18FFDG-PET in locating foci of infection, it can be very useful in aid to the diagnosis of septic foci in fever of unknown origin, a problem that always offers great difficulties and challenges for clinicians and surgeons. In cases of fever of unknown origin, Bleeker-Rovers *et al* .^27^ showed that none of the patients with periodic fever was diagnosed with focal abnormalities when the 18F-FDG-PET was normal. The negative predictive value was 100% and 18F-FDG-PET was useful in 50% of patients with a final diagnosis. When 18F-FDG-PET was added to the diagnostic protocol in patients, the number of additional unnecessary tests was significantly reduced^27^ . In addition, 18F-FDG-PET can become a useful tool to assess the effect of treating infectious and inflammatory processes that cannot be safely visualized by conventional techniques. The results of this study allow us to state that the 18F-FDG-PET can be considered very effective in the diagnosis of abdominal sepsis, as it has been in the diagnosis and follow up of cancer. Research in databases allows to state that this is the first study evaluating the accuracy of miroPET in the diagnosis of abdominal sepsis in an experimental model. The findings of the present study may have significant therapeutic implications in the clinical setting. The results encourage the further clinical use of 18F-FDG-PET. It can be useful in assessing the effects of sepsis treatment, evaluating fever of unknowing origin, detecting images that cannot be observed accurately by other imaging techniques, specially tomography, the standard exam in abdominal sepsis.

## Conclusions

The diagnostic accuracy of 18f-FDG-PET in rats with abdominal sepsis was significantly high. It was also demonstrated the high predictive ability of the abdomen/liver SUV ratio, making it easier to locate abdominal sepsis.

## Footnotes

1 Research performed at Nucleus of Experimental Surgery, Department of Surgery, Universidade Federal do Rio Grande do Norte (UFRN), Natal-RN, Brazil. Part of PhD degree thesis, Postgraduate Program in Health Sciences. Tutor: Aldo Cunha Medeiros.
